# Analysis and evaluation of environmental tobacco smoke exposure as a risk factor for chronic cough

**DOI:** 10.1186/1745-9974-3-6

**Published:** 2007-05-02

**Authors:** Beatrix Groneberg-Kloft, Wojciech Feleszko, Quoc Thai Dinh, Anke van Mark, Elke Brinkmann, Dirk Pleimes, Axel Fischer

**Affiliations:** 1 Division of Allergy Research, Charité – Universitätsmedizin Berlin, Free University and Humboldt-University, D-13353 Berlin, Germany; 2 Department of Pediatric Pneumology and Allergy, The Medical University Children's Hospital, PL-01-184 Warsaw, Poland; 3 Department of Medicine, Charité – Universitätsmedizin Berlin, Free University and Humboldt-University, D-13353 Berlin, Germany; 4 Institute of Occupational Medicine, University zu Lübeck, D-23538 Lübeck, Germany; 5 Department of Prevention, Norddeutsche Metall-Berufsgenossenschaft, D-30173 Hannover, Germany

## Abstract

Exposure to environmental tobacco smoke (ETS) and active tobacco smoking has been shown to increase symptoms of bronchial asthma such as bronchoconstriction but effects on other respiratory symptoms remain poorly assessed. Current levels of exposure to tobacco smoke may also be responsible for the development of chronic cough in both children and adults. The present study analyses the effects of tobacco smoke exposure as potential causes of chronic cough. A panel of PubMed-based searches was performed relating the symptom of cough to various forms of tobacco smoke exposure. It was found that especially prenatal and postnatal exposures to ETS have an important influence on children's respiratory health including the symptom of cough. These effects may be prevented if children and pregnant women are protected from exposure to ETS. Whereas the total number of studies adressing the relationship between cough and ETS exposure is relatively small, the present study demonstrated that there is a critical amout of data pointing to a causative role of environmental ETS exposure for the respiratory symptom of cough. Since research efforts have only targeted this effect to a minor extent, future epidemiological and experimental studies are needed to further unravel the relation between ETS and cough.

## Introduction

Environmental tobacco smoke (ETS) or passive smoking, has been demonstrated to be causally associated with a large number of human diseases although the evidence is sometimes conflicting and the tobacco industry has probably tried to cover up research data over the past 30 years as suggested recently [1]. With regard to the different diseases and symptoms associated with exposure to ETS, reliable evidence has been provided that exposure to ETS is linked with impaired lung function and aggravation of asthma in childhood and adulthood [2]. Asthmatic children with mothers who smoke were found to have more severe asthma when compared with children of non-smoking mothers. Although, parental smoking has not consistently been reported to correlate with the risk of allergic sensitization in children, it has been suggested that maternal smoking during pregnancy or exposure of children to ETS might lead to asthma via an increase of bronchial hyperreactivity, increased sensitivity to allergens, alterations in circadian variations of pulmonary function, and irritant effects [3,4]. Together, these mechanisms might be directly dependent on an increased inflammatory burden of the upper and lower respiratory tract due to an activation of neuroinflammatory reflexes, recruitment of inflammatory cells, and proinflammatory mediator release [5].

Whereas there seems to be a clear link between lung cancer and exposure to tobacco smoke [1], other respiratory symptoms such as mucus secretion or chronic cough have not been analysed in detail for the influence of tobacco smoke exposure. The symptom of cough is one of the most difficult respiratory symptoms to treat and only a little is known about the exact mechanisms in children and adults [6-8]. Pathophysiologically, coughing is coordinated by neuronal reflexes in order to protect the respiratory tract from noxious exogenous substances such as tobacco smoke or other factors [9-19] and numerous complex mechanisms underlie this phenomenon [20,25]. Recently, transient receptor potential vanilloid-1 has been suggested to play a major role in the pathophysiology of the cough reflex [26,27] and numerous research efforts have been undertaken to optimize the diagnosis and treatment of the symptom [28-35].

Whereas exposure to ETS has been demonstrated to be associated with the occurrence of numerous pathological conditions, the link between ETS and the symptom of cough has not been analysed in great detail so far. Therefore, the present study aimed to analyse the association of ETS and cough on the basis of database searches and existing clinical and experimtenal studies [36,20-58].

## Methods

Database searches were conducted using terms including "cough", "environmental", and various other terms related to tobacco smoke exposure. (date: 2006-03-03). The PubMed system was used. This is a service of the U.S. National Library of Medicine. It includes over 16 million citations from MEDLINE and other life science journals for biomedical articles back to the 1950s. PubMed includes links to full text articles and other related resources [59].

To further delineate the impact of research focussed on the symptom cough and its relation to tobacco smoke, all publication years dating back to 1969 were screened. The number of entries related to the terms tobacco and cough were assessed. Also, the different publication dates of the articles were analysed. Finally, articles were screened for their contents and relevant data was analysed.

## Results and Discussion

### Frequency of research related to cough and tobacco smoke exposure

**Figure 1:**
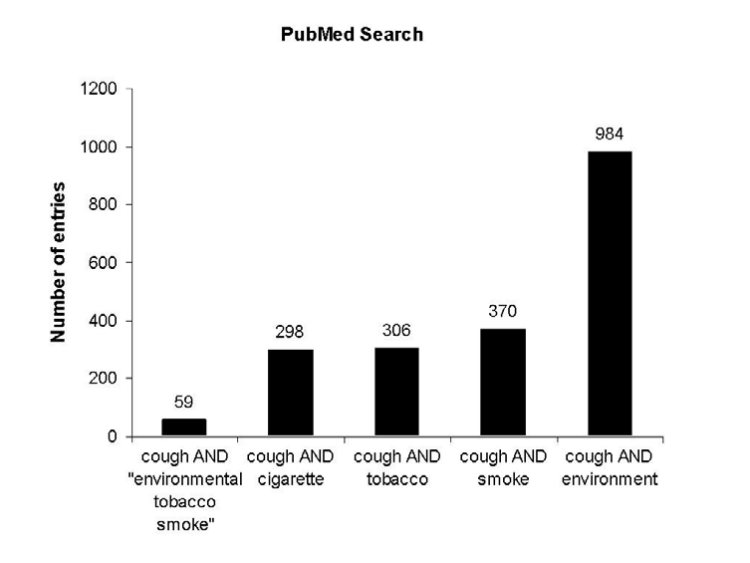
PubMed search for the terms cough and tobacco smoke exposure.

**Figure 2:**
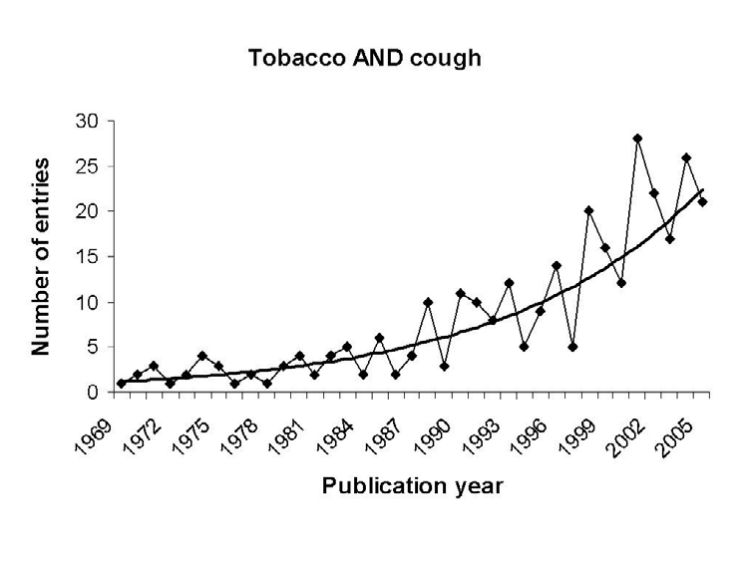
PubMed search for the terms cough and tobacco and publication dates. An exponential trendline indicates the increase over the time.

For the terms "cough" and "environment" a total of 984 entries were found in PubMed. While 370 entries contained the terms "cough" and "smoke" (Fig. 1), 306 entries contained "cough" and "tobacco" and 298 entries "cough" and "cigarette". Narrowing the research to ETS by using the terms "cough" and "environmental tobacco smoke" only 59 entries were found, suggesting that the relation of the symptom cough to ETS has not been focussed on in detail over the past few years. To further analyse the distribution of studies addressing cough and its relation to tobacco smoke, different publication dates were screened and a differential distribution was found. In this respect, an increasing frequency was found beginning from the year 1969 with one article increasing to 21 articles in the year 2005 (Fig. 2).

### Environmental tobacco smoke

There has been increasing evidence that the exposure of adults and children to environmental tobacco smoke (ETS) is a general health hazard and exerts major deleterious effects on the cardio-respiratory system [2,36,60,4,66]. In this respect, significant associations have been found between ETS for respiratory diseases such as adult and pediatric asthma. Also a series of epidemiological analyses on parental smoking and respiratory health in children have been performed [60,64,4-71]. Overall there was a very consistent picture with odds ratios for respiratory illnesses and symptoms between 1.2 and 1.6 for either parent smoking, the odds usually being higher in pre-school than in school aged children. For cough, the odds ratio when both parents were smokers was 1.67 (1.48 to 1.89) [67-71].

To assess the number of studies linking cough and tobacco smoke exposure, different search terms were used and it was found that while there was a relatively high number of studies with both the terms "cough" and "smoke" (370 entries) or "cough and tobacco (306 entries), only 59 studies had both the terms "cough" and "environmental tobacco smoke".

Important methodological issues arise in the assessment of associations between ETS exposure and respiratory diseases such as chronic cough, as there is the possibility of confounding by variables related both to the exposure and the outcome of interest [60]. By analyzing available data self-reported health conditions such as chronic cough can be related to ETS exposure in men (Table 1) and women (Table 1) who were never smokers of any tobacco products. Significant values were found for the association of chronic cough to heavy (> 40 hours/week) ETS exposure at home in men, for the association of chronic cough to heavy (> 40 hours/week) ETS exposure in small spaces, total ETS exposure, and large indoor areas in men. For women, significant values were found for heavy ETS exposure in small spaces and in large indoor areas and for total ETS exposure [60].

**Table 1:** Association between ETS exposure abd self-reported chronic cough in man.

	ETS exposure (hours/week)					
n = 514 male					Odds ratio (95% CI)	

	0	1–9	10–39	>40	Heavy vs. no exposure	Any vs. no exposure

at home	3.0	2.9	4.3	4.3**	1.33 (0.80, 2.08)	1.11 (0.89, 1.38)
Small spaces	2.8	3.2	3.4	4.7***	1.72 (1.23, 2.36)	1.25 (1.04, 1.05)
Large indoor areas	2.8	3.1	4.2	3.7*	1.26 (0.78, 1.94)	1.20 (0.99, 1.45)
Total exposure	2.7	2.9	3.3	4.4***	1.60 (1.22, 2.10)	1.22 (1.00, 1.49)

Entries were age-adjusted per 100 individuals by level of ETS exposure. * linear trend < 0.05, ** < 0.005, < 0.0001. Odds ratios were adjusted for age, alcohol consumption, body mass index, diabetes, ethnicity, education status, hypertension, marital status, physical activity at work, serum total cholesterol, and individual occupational hazards. Standard deviation (SD) for home exposure = 19.9 hours/week; SD for small spaces exposure = 15.5 hours/week; SD for large indoor areas exposure = 13.4 hours/week; SD for total exposure = 24.7 hours/week. Modified from [60].

Other data describing the effects of ETS often used nonsmoking wives and smoking discordant husbands. Here, it was also shown that wives with never-smoked husbands had lower frequencies of chronic cough, next to a better socio-economic status and better indices of the family cohesiveness. These differences were largest when comparing wives of never-smoked vs. heavily smoking husbands (more than 20 cigarettes/day), suggesting a dose-response [72].

The association between the smoking status and the prevalence of chronic cough was also analyzed in the long-term ambient air pollution and respiratory symptoms in adults study (SAPALDIA) [73]. This cross-sectional study in random population samples of adults in Switzerland reported prevalences of chronic cough in percent by smoking status and found 3.3 (2.8 – 3.8, 95% confidence interval) of never smokers (n = 4.229), 3.0 (2.3 – 3.7) of former smokers (n = 2.175) and 9.2 (8.2 – 10.2) of current smokers (n = 3.232) [73].

ETS exposure has been shown to increase symptoms of allergic bronchial asthma, but direct effects on the expression of inflammatory markers have not been analysed in detail previously. Therefore, a recent study assessed the correlation of ETS exposure with the expression of inflammatory mediators in airway secretions of children with asthma. IFN-gamma and IL-12, as well as IL-5 and IL-13 were analysed in allergic asthmatic children and healthy children [74]. Using nasopharyngeal aspiration, airway secretions were collected from 24 atopic children with asthma (age, 6–16 years) and 26 healthy control subjects, and cytokine concentration was determined by means of immunoenzymatic methods. It was shown that IL-13 levels were highly increased in patients with asthma (P < .005). Also, a positive correlation between IL-13 levels and serum IgE concentrations (r(s) = 0.55) was found in children with allergic asthma. Parental tobacco smoking lead to a significant increase in airway IL-13 secretion compared with nonexposed children and healthy control subjects (median, 860 pg/mL vs 242 pg/mL and 125 pg/mL, respectively) [74]. Together, these results indicate that ETS exposure augments the expression and secretion of IL-13 in allergic asthma. Measurements of IL-13 in secretions might be taken into account as a noninvasive marker of airway inflammation and to assess the detrimental effects of ETS.

Regarding the mechanisms between smoke exposure and chronic cough, recent studies have assessed capsaicin responsiveness [75-77]. It was shown that cough reflex sensitivity is enhanced soon after smoking cessation. Therefore, it was suggested that diminished cough sensitivity in smokers results from chronic cigarette smoke-induced desensitization of airway cough receptors. In a further study, the cough reflex sensitivity to capsaicin (C(5)) was evaluated in 11 chronic smokers who had discontinued smoking for at least 2 weeks, and then resumed smoking. It was shown that two weeks after smoking cessation there was a significant enhancement of cough reflex sensitivity; mean (+/-SEM) log C(5) decreased from 1.77+/-0.18 to 1.47+/-0.14 (p = 0.01). The subjects resumed smoking after 2–12 weeks of abstinence and a repeat capsaicin cough challenge was performed 14–23 days after resumption of smoking. The mean log C(5) increased compared to the last value obtained during the smoking cessation period (1.42+/-0.15 vs. 1.77+/-0.16, p = 0.0004) and the mean log C(5) after resumption of smoking returned to almost exactly the baseline value. These data point to a dynamic phenomenon in the sensitivity of airway cough receptors. In this respect, they seem to be promptly affected and modulated by the presence or absence of cigarette smoke [77]. Similar results were demonstrated in experimental animal models of passive ETS exposure [78].

The different reports clearly indicate an association between ETS and chronic cough. Whereas active tobacco smoking is without a doubt related to chronic cough and other more prominent diseases such as bronchial carcinoma, passive ETS may also be a relevant risk factor for chronic cough [36]. The present analysis concerns environmental exposure to tobacco smoke. In this respect, it neglects chronic cough in active tobacco smokers. Issues related to active smoking that need to be analysed in the future are the association of active tobacco smoking on the symptom of chronic cough. This is of major interest because it needs to be determined whether cough or sputum production are predictors of COPD. This association may also be assessed by studies that focus at symptom modification after smoking cessation. It will also be important to address whether it is the active exposure or the resulting damage that is causing cough in chronic tobacco smokers.

### Suspended particulate matter

A further important environmental factor contributing to chronic cough is displayed by suspended particulate matter (SPM) [36] and tobacco smoke products also belong to SPM fractions. 6 entries in the PubMed were found that linked cough to the term "particulate". Effects of SPM critically depend on the particle size and the concentration and may fluctuate with daily fluctuations in the PM10 or PM2.5 levels. The actual relationship between PM10 or PM2.5 exposure and health effects has been shown to be linear at concentrations below 100 micrograms/m^3 ^and currently there are no threshold known below which no effects occur. Large variance in short-term health effects have been reported which may base on the influence of concomitant gaseous pollutants.

A study on the association between the indoor PM10 and chronic cough in 3,709 Chinese adults demonstrated highly significant differences between study areas in the prevalence of chronic cough. Median indoor concentrations of PM10 were much higher in Beijing (557 micrograms/m^3^), where the highest prevalence for chronic cough was also found [79].

In the Swiss SCARPOL study, reported symptom rates of chronic cough, nocturnal dry cough, and bronchitis, adjusted for individual risk factors, were positively associated with PM10, NO2, and SO2. In this study, the strongest relationship was observed for PM10 (adjusted odds ratio) between the most and the least polluted community with a value of 3.07 (95% CI: 1.62 to 5.81) for chronic cough [80].

Concerning ambient air pollution, a comparison of nonsmoking residents of lower- and higher-pollution zones, which were stratified according to socioeconomic levels and sex, showed that chronic cough but not wheeze was significantly more common in the higher-pollution zone in only some of the strata [81].

## Conclusion

Environmental tobacco smoke exposure does not only lead to lung cancer and cardiorespiratory disease but is also related to numerous respiratory symptoms. Whereas it has been known for a long time that exacerbations of bronchial asthma and chronic obstructive pulmonary disease might be aggravated by ETS exposure, only little knowledge has been accumulated concerning the relation of ETS and symptoms such as cough. The present study assessed this relation by analyzing the existing literature. The results clearly indicate that ETS exposure may lead to the symptom of cough. Since only a few experimental approaches exist to date to treat cough, further studies on the clinical, experimental and molecular basis are needed. These studies should analyze the pathophysioloical basis and identify genetic factors of chronic cough. Also, new options to treat this respiratory condition need to be evaluated.

**Table 2:** Association between ETS exposure abd self-reported chronic cough in women.

	ETS exposure (hours/week)					
n = 808 female					Odds ratio (95% CI)	

	0	1–9	10–39	>40	Heavy vs. no exposure	Any vs. no exposure

at home	2.9	3.2	4.2	3.0	0.93 (0.65, 1.28)	1.14 (0.97, 1.34)
Small spaces	2.8	3.2	4.1	3.4*	1.17 (0.89, 1.51)	1.17 (1.01, 1.37)
Large indoor areas	2.9	3.1	3.6	5.1***	1.68 (1.17, 2.34)	1.12 (0.96, 1.30)
Total exposure	2.7	2.8	3.8	3.3**	1.14 (0.92, 1.42)	1.12 (0.96, 1.32)

Entries were age-adjusted per 100 individuals by level of ETS exposure. * linear trend < 0.05, ** < 0.005, < 0.0001. Odds ratios were adjusted for age, alcohol consumption, body mass index, diabetes, ethnicity, education status, hypertension, marital status, physical activity at work, serum total cholesterol, and individual occupational hazards. Standard deviation (SD) for home exposure = 19.9 hours/week; SD for small spaces exposure = 15.5 hours/week; SD for large indoor areas exposure = 13.4 hours/week; SD for total exposure = 24.7 hours/week. Modified from [60].
